# Does B Cell Follicle Exclusion of CD8+ T Cells Make Lymph Nodes Sanctuaries of HIV Replication?

**DOI:** 10.3389/fimmu.2019.02362

**Published:** 2019-10-09

**Authors:** Sarah E. Beck, Rebecca T. Veenhuis, Joel N. Blankson

**Affiliations:** ^1^Department of Molecular and Comparative Pathobiology, Johns Hopkins University School of Medicine, Baltimore, MD, United States; ^2^Department of Medicine, Johns Hopkins University School of Medicine, Baltimore, MD, United States

**Keywords:** CD8+ lymphocytes, HIV, lymph nodes, B cell follicle sanctuary, reservoir

## Abstract

As we learn more about the HIV latent reservoir, we continue to discover that the viral reservoir is more complicated than just a pool of infected resting memory CD4+ T cells in peripheral blood. Evidence increasingly points to both certain tissues and certain types of cells as potential viral reservoirs. T follicular helper cells (T_FH_) are prime targets of HIV infection—this creates a sanctuary for infected cells because CD8+ T cells generally do not enter lymph node follicles unless they express CXCR5, and are not as effective at killing infected CD4+ T cells as peripheral CD8+ T cells. In this review, we summarize the current state of research on T_FH_ cell infection in peripheral lymphoid tissues and focus on the question of whether CD8+ T cell exclusion from B cell follicles is responsible, at least in part, for establishing secondary lymphoid tissue B cell follicles as an anatomic site of HIV transcription and replication.

## Introduction

As we approach the 5th decade of the HIV pandemic and after years of intensive cure study, we continue to find a cure for HIV outside the reach of natural immunity due to the persistence of the latent reservoir found in resting memory CD4+ T cells. Retrovirus replication has several unique properties that have made eradication a challenge. Recent evidence suggests that the HIV reservoir is established in the very early stages of HIV infection (1, 2). As CD4+ T cells are the preferred target of HIV, a massive depletion of memory CD4+ T cells occurs in acute HIV infection but not all infected cells die (3–5). Because CD4+ T cells can transition to relatively transcriptionally inactive memory cells, HIV is able to “hide” from the immune system in these cells for long periods of time. In addition, because of a lack of ongoing viral replication, suppressive antiretroviral therapy (ART) does not effectively target latently infected CD4+ T cells and a study by Siliciano et al. estimated that it will take more than 73 years for an HIV-infected individual to clear the latent reservoir with ART alone (6).

Most studies of the latent reservoir have been done exclusively using peripheral blood mononuclear cells (PBMCs). This presents a problem for accurate estimation of the size of the reservoir as many lymphocytes in the body reside in the lymphatic system and tissues. Therefore, throughout the body there are likely other reservoirs and sanctuaries for HIV which may have different characteristics from the peripheral blood reservoir (7).

### CD8+ T Cell Immunity Is Critical to Controlling HIV Infection

Studies in people living with HIV (PLWH) and *Simian immunodeficiency virus* (SIV)-infected primates support the hypothesis that CD8+ T cell immunity is critical to natural control of HIV infection. An early HIV-specific CD8+ T cell immune response is associated with increased viral control compared to patients that lack an early cytotoxic T lymphocyte (CTL) response (8, 9). SIV-infected macaques that are pharmacologically depleted of CD8+ T cells go on to develop higher viremia and more rapidly progressive disease compared to those SIV-infected macaques that are not CD8+ depleted, providing more evidence for the importance of CD8+ T cell-mediated HIV control (10–12).

A small subset of PLWH are able to control viral levels below the limit of detection in the absence of ART (13, 14). Elite Controllers (EC) are individuals that maintain a viral load below 50 copies of HIV-1 and extremely rare (<1% of the HIV infected human population). ECs have provided a great deal of insight as to the importance of CD8+ T cells in naturally controlling HIV disease progression (13). Certain human leukocyte antigen (HLA) alleles, such as HLA-B^*^57 and HLA-B^*^27 are significantly overrepresented in ECs (15, 16). Since T cell immunity is HLA allele restricted, this provides compelling evidence of the importance of CTL-mediated control of HIV replication. On a population scale, viral CTL escape mutations track along with expression of certain HLA alleles (17), demonstrating that HIV has developed a crucial mechanism of immune evasion via the development of CTL escape mutations. Additionally, multiple studies have also shown that the quality of CD8+ T cell response is associated with viral control in ECs (18–21).

Despite the importance of CD8+ CTL-mediated control of viral replication in ECs, CTLs alone are incapable of completely eliminating HIV and reservoirs of replication-competent virus are present in these subjects (22). Bailey et al. sequenced plasma virus and peripheral CD4+ T cell proviral DNA from HLA-B^*^57 ECs and found a striking discordance in sequences present in the HLA-B^*^57 restricted epitopes (23). Escape mutations were rare in CD4+ T cells but present in every single plasma virus sequenced. This suggested that CD8+ T cells were exerting strong selective pressure in these patients and that the plasma virions were not being produced from peripheral CD4+ T cells. This led to two question; how is HIV able to still replicate in the face of effective CTL immunity in these subjects? And where is this viral replication occurring? In this review, we hope to explore some answers to these questions as they will be important to understand if we are to develop CTL-mediated strategies to induce HIV remission in patients with progressive disease on ART.

### Follicular Tissue as a Sanctuary Site for HIV Replication

As we learn more about the HIV latent reservoir, we continue to discover that the viral reservoir is more complicated than just infected resting memory CD4+ T cells in peripheral blood. Evidence increasingly points to both certain tissues and certain types of cells as potential sites of latent reservoir maintenance. There is evidence that multiple tissues, including the brain (24–26), spinal cord (27), and reproductive organs (28, 29) could be sanctuary sites for HIV, possibly because of their immune privileged status. Other tissues, such as the spleen, lung, and adipose tissue have also been suggested as sites of HIV persistence (30–32).

However, secondary lymphoid tissue is likely one of the largest potential sites for HIV replication and persistence throughout the course of infection (33–36).

Some studies suggest that HIV continues to replicate in lymphoid tissues in PLWH on fully-suppressive ART regimens, albeit at a lower level than untreated viremic individuals (36) and data suggestive of ongoing replication have also been seen in studies using ART-treated SIV-infected macaques (37, 38). However, other studies have not found evidence of ongoing viral replication in lymphoid tissue and have suggested that HIV is maintained by clonal expansion of infected CD4 T cells in LN tissues rather than ongoing viral replication (39). Regardless of the mechanism, it is clear that a potential reservoir exists in lymphoid tissue and the inability of the immune system to eliminate these infected cells needs further investigation.

While primary lymphoid tissue, the bone marrow and thymus, are considered the birthing sites for T and B cells, it is within secondary lymphoid tissues that activation and lineage maturation occur. Secondary lymphoid organs include the spleen, tonsils, and adenoids, bronchiolar-associated lymphoid tissue (BALT), gut-associated lymphoid tissue (GALT), and all lymph nodes. Secondary lymphoid tissues contain large numbers of CD4+ lymphocytes susceptible to infection, and recent studies continue to support the hypothesis that follicles within secondary lymphoid tissues represent a major site of viral persistence. Using inguinal lymph nodes collected from viremic HIV patients, Folkvord et al. showed that CD4+ T cells within follicles are 31 times more likely to be positive for HIV RNA than CD4+ T cells in extrafollicular sites (35). The same group later showed that CD4+ T cells within follicles are 40 times more likely to produce HIV RNA compared to extrafollicular tissues (40).

Over the last several decades, multiple studies in primates have also provided compelling evidence that lymphoid follicles are a focal point for SIV replication in chronic infection. By morphologic studies using *in situ* hybridization, there are consistently higher concentrations of SIV+ RNA in follicular than extrafollicular zones in multiple secondary lymphoid organs, including peripheral lymph nodes, the spleen, and GALT in multiple sites (41, 42). Early studies pointed to follicular dendritic cells (FDCs) as the major source of maintenance of HIV in B cell follicles of secondary lymphoid tissues (43, 44), and it certainly seems likely that these cells play a major role in infecting CD4+ T helper cells in secondary lymphoid tissue, contributing to CD4+ depletion and immunodeficiency (45). However, in the presence of undetectable viral loads, FDCs would not be capable of continuously infecting CD4 T cells, suggesting that the latent reservoir is mostly comprised of quiescently infected CD4+ helper T cells in lymphoid follicles. Therefore, it has been proposed that CD4 T cells can become infected outside of the lymph node prior to entering the follicle and subsequently differentiating into T_FH_. Regardless, all mechanisms point to one central issue that remains: the immune system is not capable of acting on infected cells within the LN.

### Follicular Helper T Cells in Secondary Lymphoid Tissue as a Viral Reservoir

**Figure 1:**
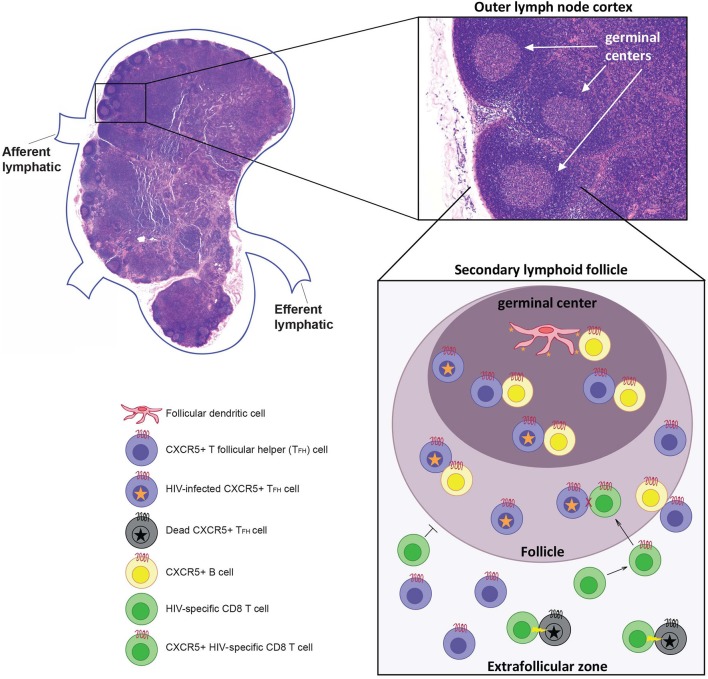
Afferent lymph fluid enters at the cortex of the node, and efferent lymph fluid leaves at the hilus through lymphatic vessels. The lymph node (LN) itself consists of immune cells of both lymphocytic and myeloid lineage arranged on an interlacing lymphoreticular network that organizes these immune cells, promoting activation and clonal expansion (46). The LN cortex is divided into an outer cortex, containing primary and secondary lymphoid follicles, and an inner cortex. Primary follicles consist mostly of B cells and do not contain a germinal center, while secondary follicles, which form after immune activation, consist of a mantle of B cells surrounding a central germinal center consisting of centroblasts and follicular dendritic cells (FDCs). T follicular helper (T_FH_) cells can be found in the extrafollicular zone (T cell zone) and B cell follicles. T_FH_ cells are a unique CD4+ T cell subset within secondary lymphoid tissues that interact with B cells at the T:B zone. They are characterized by high expression of the B cell follicle-homing chemokine receptor CXCR5, allowing T_FH_ cells to migrate to the site of T cell-mediated B cell activation in secondary lymphoid tissues (47–49). Follicular dendritic cells (FDC) can present infectious HIV virions for long periods of time which leads to the infection of T_FH_ within the germinal center (50). It is also possible that CD4 T cells are infected outside the follicle and differentiate into T_FH_ within the follicles. Infected T_FH_ cells are primarily located within B cell follicle and germinal centers because CTLs cannot enter these regions unless they express CXCR5. If CTLs do express CXCR5 they are able to enter the follicle but have been shown to be incapable of killing HIV infected T_FH_ cells. However, infected T_FH_ that leave the follicle can be killed by HIV-specific CTLs.

How has HIV been able to “hide” in lymphoid tissues so effectively? Much of the answer likely lies with the follicular helper T cell subset (T_FH_ cells), located within germinal centers of secondary lymphoid tissues. A brief review of lymph node (LN) structure and function is provided in Figure 1 to help illustrate how T_FH_ cells may contribute to the reservoir. LNs are secondary lymphoid tissues that are dispersed throughout the body along the lymphatic vasculature. Since they number in the hundreds, these are relatively easy sites of secondary lymphoid tissue to surgically biopsy, and so they are some of the most commonly studied secondary lymphoid tissue in HIV infection.

T_FH_ cells are a unique CD4+ T cell subset within secondary lymphoid tissues that interact with B cells at the T:B zone. They are characterized by high expression of the B cell follicle-homing chemokine receptor CXCR5, allowing T_FH_ cells to migrate to the site of T cell-mediated B cell activation in secondary lymphoid tissues (47–49). CD4+ CXCR5+ T_FH_ cells have also been found in the peripheral blood, although it is not yet clear if these cells are really the same population as the follicular T_FH_ cells or if they represent a completely different T_FH_ population, and there remains debate as to their significance is in respect to the latent reservoir (47, 51, 52). Recent evidence even suggests that a proportion of circulating T_FH_ cells originate in lymph nodes and subsequently traffic to the periphery via the lymphatics (53).

Using lymph nodes isolated from HIV viremic individuals, Perreau et al. previously showed that T_FH_ cells in lymph nodes are a potential sanctuary for viral replication within the CD4+ T cell compartment. By flow cytometry, follicular T_FH_ cells were identified as the largest subcategory of CD4+ T cells to be positive for HIV DNA, and *in vitro* these cells were more permissive to HIV infection than other CD4+ T cell subsets (54). These studies were in concordance with data collected from SIV-infected rhesus macaques (38, 42). Recent studies have found higher levels of inducible virus and cell-associated HIV-1 RNA in T_FH_ cells than in other memory cells in both viremic subjects and patients on long-term ART and concluded that there is ongoing viral transcription in these cells (55, 56). The mechanism for ongoing transcription was unclear but some studies have found reduced concentrations of antiretroviral drugs in lymph nodes cells in humans (57) and in NHPs (58). However, in contrast to these studies, a recent study found that the concentration of antiretroviral drugs in lymph nodes in humans and NHPs exceeded that found in plasma (59).

Studies utilizing both native and non-native SIV hosts have been instrumental in supporting that T_FH_ cell infection in germinal centers plays a major role in maintaining the viral reservoir. Native host species of SIV, such as the sooty mangaby, develop chronic viremia but do not develop immunosuppression and progression to AIDS (60, 61). Brenchley et al. demonstrated that untreated sooty mangabys and rhesus macaques (a non-native host for SIV) both have HIV-infected CD4+ T_FH_ cells within lymph nodes, but that rhesus macaques, as non-native hosts for SIV that develop progressive disease, have far more infected T_FH_ cells compared to the non-progressor sooty mangabys (42). This suggests that the level of CD4+ T_FH_ cell HIV infection is a critical difference between non-progressive, native SIV host species and progressive, non-native macaques, but the immunologic mechanism behind this difference is unclear.

Similar to human ECs, there are a small proportion of SIV-infected macaques that naturally control infection, and, like human ECs, certain MHC class I alleles are overexpressed in these animals (62, 63). Fukazawa et al. recently demonstrated that SIV infection was restricted to CD4 T_FH_ cells within the lymphoid follicle of SIV-infected EC macaques but not progressor macaques (38). They also showed that viral RNA in CD4+ T_FH_ cells was higher than non T_FH_ CD4+ memory cells in EC macaques (38), further supporting for the theory that T_FH_ cells within the lymphoid follicle represent a potential reservoir for HIV and SIV latency. Boritz et al. also found evidence of ongoing viral replication in lymphoid tissue in untreated PLWH who had low level viremia (64). Sequence analysis revealed that the reservoir in lymphoid tissue was distinct from the reservoir in peripheral CD4+ T cells suggesting that lymphoid tissue may be the source of the plasma escape mutants seen in ECs in prior studies (24).

### CD8+ T Cell Exclusion From Follicles

Why does HIV continue to replicate in follicular T_FH_ cells even with robust and effective CD8+ T cell mediated viral control? It is likely that CD8+ T cells are, for a variety of possible reasons, unable to control HIV replication in T_FH_ cells, but the exact mechanisms of this CTL failure are still unclear. However, there are now several proposed mechanisms for why CD8+ T cells may be unable to effectively target HIV-infected follicular CD4+ cells. Lymphocytes in secondary lymphoid tissues are phenotypically and functionally distinct from their counterparts found in peripheral blood (65), and these different effector functions that may also extend to CD8+ T cell cytotoxicity. In fact, CTLs have been shown to have altered cytolytic activity in lymphoid tissue, potentially reducing their effectiveness in eliminating HIV-infected CD4 cells in follicles (66). Compared to their peripheral blood counterparts, CD8+ T cells within secondary lymphoid tissue have altered expression of cytolytic markers, including dysregulated and low expression of the granzyme B and perforin (66). However, this conclusion is somewhat controversial and other studies have suggested that these lymphoid CD8+ T cells are functional by cytolytic assays (40).

One hypothesis that could explain the lack of CD8+ T cell control of low level viral replication in germinal centers is the theory that B cell follicles represent immunologically privileged sites due the exclusion of CD8+ T cells. While one study showed that PLWH have more CD8+ T cells in lymph nodes than HIV negative subjects (67), multiple groups have shown that CD8+ T cells fail to accumulate at the site of infected T_FH_ helper cells in chronic HIV infection and both acute and chronic SIV infection (38, 40, 68, 69). And it has been shown that the lack of the B cell follicle homing marker CXCR5 on many CD8+ T cells exclude these CTLs effectively from B cell follicles, preventing CTLs from effectively killing HIV-infected T_FH_ cells in follicles (38, 40). Early work assessing lymph node biopsies untreated PLWH suggested that CD8+ T cells failed to specifically localize adjacent to HIV-producing cells in lymphoid tissues (69). Using lymph node biopsies from HIV viremic patients, Connick et al. showed that, although there are a large number of HIV-specific tetramer positive CD8+ T cells in the lymph node, these cells were largely not present in lymphoid follicles where HIV is predominantly replicating in CD4+ cells (40). More recently, a study by Fukazawa et al. confirmed that not only is SIV infection is restricted largely to T_FH_ cells in GCs of SIV-infected rhesus macaque ECs, but the depletion of CD8+ cells relocated HIV-production to non-T_FH_ cells within lymphoid tissue (38). Once CD8+ cells recovered, SIV-infection was once again restricted to T_FH_ cells within GCs of lymphoid tissue (38). These studies provide compelling support for the hypothesis that B cell follicles are indeed sanctuaries for HIV replication, and that this sanctuary is largely mediated by the inability of CD8+ T cells to effectively kill T_FH_ in sequestered in GCs.

Although the studies mentioned have shown CD8+ T cell exclusion in chronic infection, until recently, there was little work looking at follicular CD8+ T cell exclusion and killing of T_FH_ cells in acute infection. Using SIV-infected rhesus macaques, Li et al. has shown that CD8+ T cell exclusion from follicles appears to start as early as several weeks after infection (68).

### CD8+ T Cell Functionality in Lymphoid Follicles

However, CD8+ T cell exclusion from B cell follicles may not be the only explanation for the maintenance of an HIV reservoir in lymphoid tissues, as there is conflicting data as to how much CD8+ T cell-exclusion from the B cell follicles actually occurs. Several studies have demonstrated a population of CXCR5 + CD8+ T cells within B cell follicles. These cells have been shown to have different transcriptional profiles from their CXR5-CD8+ T cell counterparts and their role in controlling viral replication has been the subject of many recent studies (70, 71). It has been shown that increased CXCR5+ effector CD8+ T cells in secondary lymphoid tissue is associated with increased control of chronic viral infections, including HIV (66, 72, 73).

Several groups have studied the functionality of follicular CXCR5+ CD8+ T cells in the context of lymphocytic choriomeningitis virus (LCMV) infected mice, a frequently used mouse model of chronic viral infection with some similarities to HIV infection (72, 73). These murine studies have shown that virus specific CXCR5+ CD8+ T cells become activated and migrate into B cell follicles and were able to control viral replication in chronic infection (72, 73). Similar findings have been shown in PLWH, where an inverse correlation was found between viral loads and the frequency of virus-specific CXCR5+ CD8+ T cells in the B cell follicle (72). Interestingly, in another study, there was no correlation between viral loads and granzyme B and perforin expression by CXCR5+ effector CD8+ T cells (66), raising some questions about the true killing capacity of follicular CXCR5+ CD8+ T cells. Clearly more research into this area is needed to answer the question of if follicular CD8+ T cells represent a distinct subset of CD8+ T cells that are less efficient at killing HIV-infected CD4+ T cells.

### Targeting CD8+ T Cells to HIV-Infected CD4+ T Cells in the Lymphoid Follicle

How do we reconcile the need for CD8+ T cell immune responses within B cell follicles to target HIV-infected T_FH_ cells with the possible inability of these CD8+ T cells to either access or kill infected cells within B cell follicles? There are several groups that are working on strategies to specifically direct CD8+ T cells into follicles at the site of smoldering HIV infection of T_FH_ cells. One possibly strategy uses pharmacologic chemokines to direct CD8+ T cells into the B cell follicles. The cytokine IL-15 is important for maintenance of memory CD8+ T cells, so IL-15 agonists are an attractive pharmacologic target to boost CD8+ T cell-mediated HIV-1 immunity. Webb et al. showed recently that the IL-15 superagonist ALT-803 both activates and induces migration of CD8+ T cells into B cell follicles (74). ALT-803 treatment was effective in reducing the amount of SIV RNA and DNA in B cell follicles in ALT-803 treated EC macaques (74). Watson et al. used heterodimeric IL-15 in rhesus macaques to successfully increase CD8+ T cells within lymph node follicles and decrease HIV-1 viremia (75). Taking a different approach, Ayala et al. genetically engineered rhesus CD8+ T cells to overexpress CXCR5 which resulted in increased CD8+ T cell trafficking to rhesus B cell follicles (76). Conversely, Ferrando-Martiez et al. have employed anti-HIV/anti-CD3 biphasic antibodies to redirect follicular CD8+ T cells to kill HIV-infected CD4+ T cells *in vitro* (77), indicating that, at least with the help of biphasic antibody stimulation, follicular CD8+ T cells are capable of effective killing. Based on these studies, genetic engineering of CD8 T cells or treatment with immunotherapy compounds show promise as an avenue to specifically target LN sanctuaries and reduce long-term viral reservoirs in HIV-1 infected individuals.

However, it should be noted that, immunologically speaking, having follicular CD8+ T cells capable of killing within a lymphoid follicle could lead to the disruption of the follicle and the immune response itself. It seems that the existence of a mechanism to exclude activated CD8+ T cells or prevent killing within follicles may be necessary to maintain the integrity of the immune response, but further studies in this area are sorely needed.

## Discussion

Years of HIV and SIV latency research has revealed several important conclusions, first that lymphoid tissues represent a major site for HIV replication in viremic individuals and a significant reservoir of inducible virus in patients on ART. Secondly, CD4+ helper T cells are more likely to be productively infected within lymphoid follicles compared to extrafollicular sites. And finally, cytotoxic CD8+ T cells appear to be unable to effectively target these HIV infected cells when localized to a lymphoid follicle. The mechanisms behind the inability of CD8+ T cells to kill productively infected CD4+ cells in follicles are not completely understood. There is evidence that lymphoid CD8+ T cells have deficient cytolytic capabilities compared to CD8+ T cells in peripheral blood, other studies suggest that the CD8+ T cells are unable to target HIV infected CD4+ cells in follicles because of the lack of expression of appropriate chemokine homing receptors, therefore excluding the CD8+ T cells from trafficking to follicular sites of HIV replication. Regardless of the exact mechanism, ineffective CD8+ T cell killing of HIV-infected CD4+ helper T cells in secondary lymphoid tissue follicles is a major barrier to any strategy of HIV cure that relies on CTL-mediated eradication of infected CD4+ T cells. Further studies are needed to clarify the functionality of follicular CD8+ T cells in chronic HIV infection, to discover new ways of targeting CD8+ cells effectively to a major site of ongoing HIV replication as well as to determine if CTL killing within a follicle will alter the integrity of the immune response. Hopefully these future studies will enable scientists to effectively target and eliminate an important sanctuary of infected CD4+ T cells.

## Author Contributions

SB wrote the manuscript. RV and JB reviewed and edited the manuscript.

### Conflict of Interest

The authors declare that the research was conducted in the absence of any commercial or financial relationships that could be construed as a potential conflict of interest.
